# EMB7/483: Use of the World-Wide-Web to Identify Unpublished Evidence for Systematic Reviews

**DOI:** 10.2196/jmir.1.suppl1.e25

**Published:** 1999-09-19

**Authors:** G Eysenbach, TL Diepgen

**Affiliations:** ^1^Institute for Clinical Social Medicine, HeidelbergGermany

## Abstract

**Introduction:**

One important aspect of a Systematic Review (SR) is to use a comprehensive search strategy to identify evidence such as randomized controlled trials (RCTs) from multiple sources. One particular concern is to find unpublished evidence to avoid publication bias. Traditional methods to identify RCTs which are mentioned in the Cochrane Collaboration Handbook include using Collaborative Review Group trial registers, checking reference lists, personal communication, electronic databases and hand searching. The Internet represents a potential additional source for identifying evidence for systematic reviews, but the actual use of the Internet for locating unpublished evidence has not been systematically studied.

**Objectives:**

To determine whether and to which extent RCTs and other unpublished evidence useful for systematic reviews can be identified by searching the World-Wide-Web in addition to using traditional approaches mentioned in the Cochrane Collaboration Handbook. To determine on which kind of web pages hints to unpublished RCTs could be located and to determine possible search strategies and tools for the Internet. To provide future directions for research in this area and for the development of specialised search engines.

**Methods:**

A random sample of 8 completed, recently updated Cochrane Systematic Reviews (CSR) were selected and their search strategies were adapted for the World-Wide-Web. The Internet was searched and hints to potentially relevant unpublished or ongoing trials found on Web Pages were recorded and compared to unpublished evidence used in the original CSRs.

**Results:**

Information on unpublished and ongoing trials could be found on the Internet for 4 of the 8 CSRs.

**Conclusions:**

Searches on the Internet should be routinely included in the search strategies for CRs. New tools for locating ongoing trials (a specialised search engine) should be considered. A problem is how to deal with published evidence with questionable reliability found on the Internet that has not been published in peer-reviewed journals.

